# The War and Voluntary Hospital Beds

**Published:** 1915-10-30

**Authors:** 


### THE WAR AND VOLUNTARY HOSPITAL BEDS.

### MILITARY CONVALESCENTS v. URGENT CASES.

As every social worker knows, there is usually a delay in securing the admission of a suitable but non-urgent case into the wards of a hospital, and also that there is at most institutions a waiting list of such cases. The time elapsing between registration and admission depends upon how far the hospital and the extent of its accommodation is adequate as regards the requirements of the neighbourhood it serves or the speciality it treats. If this is a familiar feature of hospital work in normal times, the difficulty must have been aggravated by the fact that since the outbreak of the war a number of beds have been placed at the disposal of the military authorities for wounded soldiers. Some of these are beds additional to ordinary numbers, which have been either added to the ward accommodation or placed in other departments temporarily lent for the purpose. But there are many others that are beds which before the war were used by the civil population. This action on the part of the executives has, no doubt, secured the approval of the governors and supporters of the hospitals, who agreed to what was intended for the use of one group of persons being used for another group, justifying the arrangement by the urgency of the situation caused by the war. The assistance was given with every patriotic impulse, but could not be regarded as anything but an endeavour to stem the tide of necessity. In every way the convenience of the military authorities was considered, the hospitals waived their traditional right to a preliminary medical inquiry into the condition of the patients, and freely offered the best at their disposal to our brave wounded soldiers.

### Existing Arrangements not the Best.

It can be argued, of course, that the soldier is in any case a suitable subject for hospital relief, but admitting this, if it is granted that sufficient time has elapsed to overcome the confused and tentative arrangements made last year, the soldier should now be received on equal terms with the civilian. It is in no carping spirit that these words are written, but more in conformity with the sentiment that the very best use should be made of the whole of our resources in every phase of national life. The essence of economy lies in making the most suitable use of resources. At the present moment the most suitable use of the resources of the voluntary hospitals is not being made. Of the military patients now occupying the wards it can be said without the least exaggeration that in respect of quite one-half of them they would not be there at all if they were civilian patients. This is not to say that they do not require skilled attention; it is only to raise the point as to how and where that skilled attention should be given. There can be but the one answer as regards many of these cases of a minor medical or surgical importance, and that is that they should receive attention either at the military hospitals and convalescent homes or in the out-patient departments of the voluntary hospitals.

It must be remembered that at the present moment every bed is far more difficult to maintain than it was twelve months ago. Practically every article in daily use in a hospital has increased enormously in cost. There is a shortage in workers attached to all departments. Medical men suited to institutional life do not apply even in small numbers for the various vacancies offered. The difficulty in obtaining new sisters, male and female nurses, and servants of all kinds is increasing every day, and the rates of remuneration of all grades are higher—in some instances to a startling extent—than formerly.

The military authorities have now had a whole year to perfect their arrangements as regards home medical treatment of the sick and wounded of the Expeditionary Forces. Moreover, during the last month or two there has been a distinct lull in the number of casualties coming from abroad, and the pressure on the offices of the Director of Medical Services and his deputies has been much relieved. It is reasonable to say that ample opportunity has been afforded to get things into proper shape, but the fact is that the methods of dealing with the wounded are in all important respects the same as they were twelve months ago.

The trouble in respect of these in-patient soldiers who should be out-patients is that they cannot live in their own homes and secure the necessary though limited attention they require, because their homes are too remote from the institutions chosen for their treatment.

### An Offer to Establish Hostels.

One of two things is needed, either to arrange to receive all the wounded at the military hospitals, and send to occupy the beds at the voluntary hospitals those only who are medically or surgically suitable. The other alternative is to institute something between the home and the hospital, some place where the soldiers can live under discipline and attend their appropriate institutions for treatment. The need of improved arrangements is obvious to most administrators of voluntary hospitals, and their opinion is quite well known in Whitehall. In view of the fact that the military hospitals have not the beds requisite to accommodate in the first instance all the cases landed in England, and do not hope to be in a position completely to fulfil their functions without outside aid for some considerable time to come, a strong committee has offered to establish hostels and manage them if the War Office will make grants on a sufficient scale for maintenance (probably lower than that in use in respect of the hospitals) and provide authority to enforce discipline. Up to the present the authorities have not accepted this offer, and appear to be quite contented with existing arrangements. The chief argument against such a scheme can only be in the question of discipline, but this does not appear to be one of such enormous difficulty as to allow it to bar the way to a reform that is undoubtedly much needed. Our wounded soldiers are worthy of the very best attention it is possible to give them, but the present uneconomical use of the voluntary hospitals is a serious injustice to the sick poor.

